# Sustainable Behavior with Respect to Managing E-Wastes: Factors Influencing E-Waste Management among Young Consumers

**DOI:** 10.3390/ijerph20010801

**Published:** 2023-01-01

**Authors:** Swati Garg, Asad Ahmad, Dag Øivind Madsen, Shahab Saquib Sohail

**Affiliations:** 1 Department of Management, School of Management and Business Studies, Jamia Hamdard, New Delhi 110062, India; 2 USN School of Business, University of South-Eastern Norway, 3511 Hønefoss, Norway; 3 Department of Computer Science and Engineering, School of Engineering Sciences and Technology, Jamia Hamdard, New Delhi 110062, India

**Keywords:** awareness, environmental concern, e-waste management, financial benefits, government policy, sustainable behaviour, theory of planned behaviour

## Abstract

With the proliferation of technological tools and the advancement in electronic devices and accessories, consumers across the world are changing and upgrading their electronic devices at an alarming rate. However, these developments have raised concerns related to electronic waste (E-waste). E-wastes contain toxic substances which may have a negative impact on both humans and the environment. This issue needs to be addressed by the research community, i.e., what would be the best way to get rid of existing devices? It is clear that countries need to work towards a more sustainable consumption pattern and consumers need to change their behaviour. The present study focuses on sustainable behaviour of consumers in terms of e-waste management. In this context, the study attempts to explore the factors influencing e-waste management among young consumers. In the present study, the Theory of Planned Behavior is extended by including the additional factors Government Policy, Environmental Concern, Financial Benefits and Awareness. A researcher-controlled sampling was employed to collect data from 524 respondents. Partial least square structural equation modelling (PLS-SEM) was used to validate the questionnaire constructs and confirm the relationships among the variables. The findings of the study suggest a significant role for government policy, financial benefits, environmental concerns, attitude, subjective norms, and perceived behavioural control in determining young consumers’ behavioural intentions toward the management of e-waste. The study findings have implications for both researchers and marketing practitioners.

## 1. Introduction

When electrical or electronic equipment (EEE) is discarded or thrown away without proper recycling, it becomes WEEE (Waste Electrical or Electronic Equipment) [1]. With a high consumption pattern of electronic items by consumers, manufacturing of these items has also risen steeply [1]. Therefore, it is necessary to focus on sustainable consumption of electronic items. It is well-known that e-waste contains toxic substances, which may have a negative impact on both people and the environment [2]. Therefore, it is important to prevent the unnecessary use of electrical equipment, which would improve the lives of present and future generations and help in protecting the environment as well.

One way to protect the environment is to focus on developing a circular economy instead of the traditional linear economy [3]. A linear economy is one in which a product is produced, and after its final use, it is disposed of. A circular economy, on the other hand, is one in which a product is used and then recycled. A circular economic model has significant economic advantages for the electronic and electrical industries. By 2030 and 2040, a circular business model for electronics could cut consumer expenses by 7% and 14%, respectively [4]. It has also been observed that non-functional goods are inaccurately categorised as ‘used goods’ and sent to lesser-developed nations in Africa and Asia by utilising a license to export of superfluous items for reuse or refurbishment [5].

It is important to focus on 4Rs of recycling: (1) Reducing waste by avoiding excessive equipment use, (2) Reusing goods to the maximum extent possible, (3) Recycling by turning waste into new products, and (4) and last but not least is Recovery of resources from waste [6]. With the changing scenarios and advancement in technology, recovery plays an important role. According to a report, three design-driven principles serve as the foundation of the circular economy: (i) Cut back on waste to reduce pollution; (ii) circulate goods and supplies (at their highest value); and (iii) restore nature [4].

Due to changing technology and upgraded features, most consumers, and in especially young consumers, are enticed towards new and upgraded versions. This persuasion of consumers ends up creating a pile of e-waste. The 4Rs can help in reducing the impact of waste by managing e-waste for the betterment of the environment. Proper recycling and reuse of e-waste not only helps the environment, but also reduces the number of precious metals (Gold (Au), Silver (Ag), Palladium (Pd), Gadolinium (Gd), Cerium (Ce), and many more [7]) used in the manufacturing of electronic products [8].

As per facts given by UNEP [9], the world might save USD120 billion annually if everyone upgraded to energy-efficient bulbs, and by 2050, if there are 9.6 billion people on the Earth, it might take almost three planets’ worth of natural resources to maintain the current standard of living. SDG (Sustainable Development Goals) Report 2020, Goal-12 which ensures “sustainable consumption and production patterns” states that e-waste increased by 38% during the period 2010–2019 [10]. This is one of the most significant reasons for all governments, both developed and developing countries [11], to pay close attention to the SDGs. However, it could also be argued that sustainability is not only the responsibility of the government or any particular organization, but everybody should take a part in shifting towards sustainable everyday activities.

Every year, the amount of e-waste generated is equivalent to the weight of 5000 Eiffel towers [12]. India generated 1,014,961 tonnes of e-waste in the year 2019–2020, which was more than the previous year 2018–2019 by 32%. It has also been reported that only 3.6% and 10% of the wastes were collected in 2018 and 2019, respectively [11]. These huge numbers are cause for concern as the planet will increasingly turn into a landfill. Over the past century, technological advances and the advent of e-products have changed in unanticipated ways. Every month, India produces more than 50,000 tonnes of e-waste. It is not surprising that the rate of e-waste generation has been increasing at a steady rate of 23.7 percent per year for the past decade. India, as the world’s third-largest producer of e-waste, must develop effective e-waste management solutions and responsibility for its own e-waste management [13]. Delhi is second only to Maharashtra in generating e-waste [14]. China, the US, and India are among the top three e-waste generating countries with 10,129, 6918 and 3230 kilotons, respectively. On the other hand, the recycling rates in China and the US are 16 and 15%, respectively, while in India it stands at an alarmingly low rate of 1% [15]. As per “The 2022 Environmental Performance Index (EPI)”, India ranks 180 out of 180 countries based on 40 indicators.

**Figure 1:**
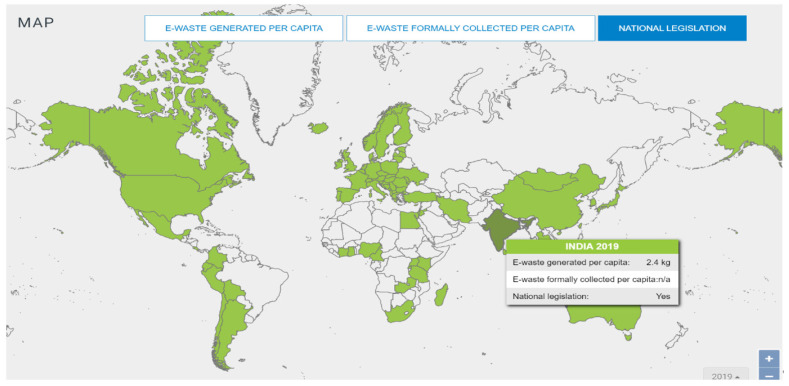
Global E-waste Statistics. Source: Global E-waste Statistics Partnership, 2019 [17].

New technological innovations and shortened lifecycles are the main reasons for this e-waste as consumers replace and upgrade their electronic devices [16]. India generated 2.4 kg per capita of e-wastes in the year 2019 [17] (Figure 1). India has 51 e-waste material collection points in 20 different states There are 400 electronic waste recyclers and dismantlers working in 20 different states of India such as Andhra Pradesh, Assam, Chhattisgarh, Gujarat. The total annual processing capacity of these licensed dismantlers and recyclers is 1,068,542.72 tonnes [18]. However, since the majority of waste is still managed by the informal sector, the official recycling capacity is underutilised [19]. The informal sector plays an important role in managing e-waste. NGOs (Non-Governmental Organization) serve as a conduit between waste pickers and electronic manufacturers under pressure to collect and recycle what they sell. In India, the world’s third largest producer of e-waste [15], 95% of consumer electronics are recycled informally [20].

Managing e-waste is a global concern these days which could harm the planet in various ways. India has one of the fastest growing economies in the world, with domestic demand for consumer durables rising rapidly [21]. With such a high growth rate, India is not lagging behind other countries in generating e-waste. However, the importance of effective e-waste management is beginning to be understood in South Asia. Despite several other nations exploring comparable legislation, India is the only country in Southern Asia having e-waste laws. Only authorised dismantlers and recyclers are allowed to collect e-waste in India due to legislation that was put in place in 2011 that governs the management of e-waste. The E-Waste (Management) Rules of 2016 included a manufacturer, dealer, refurbisher, and Producer Responsibility Organization (PRO) [19].

Therefore, in the current study, we aim to study the factors influencing e-waste management by among young consumers. There are several reasons why young consumers were chosen as respondents. Firstly, youngsters (15–34 years) [22,23] are representative of Indian society due to their high share of the population [24]. Secondly, they are considered to be more tech-savvy than other generations, which makes them early adopters of any new or upgraded product [25]. Thirdly, the younger population might be increasingly engaged in high consumption of electronic goods since they are perceived to be status symbols [26].

## 2. Proposed Model and Hypotheses Development

Several researchers have applied Theory of Reasoned Action (TRA) and Theory of Planned Behaviour (TPB) to investigate attitudes and behaviour in e-waste management [26,27,28,29,30,31]. TRA is a model from social psychology that was developed by Fishbein and Ajzen. According to the TRA, behaviour is the result of three major factors: attitudes toward behaviour, subjective norms, and behavioural intentions [27]. To overcome the limitations of TRA, Ajzen [32] developed TPB. TRA comprised attitudes and subjective norms as variables, and later Ajzen developed TPB with an additional variable of perceived behaviour control. Both the TRA and TPB models have been used extensively and are shown to be helpful in understanding behaviour, with significant contributions from perceived behavioural control [33]. Researchers have criticized the TRA model by highlighting that this model works well for the study of behaviour under volitional control (i.e., people believe that they can behave the way they want) [27,33]. There is much consumer-based research on buying intentions with respect to green marketing and customers’ green product purchasing intentions. Taking the cue from the existing literature, in the present study, we have further extended the TPB model by taking into account four additional variables, three of which have been studied in the existing literature: government policy [34], awareness [5,26,29] and environmental concern [5,27,28,34,35,36,37]. The fourth variable is financial benefits, but we were unable to find evidence in the existing literature that financial benefits influence the purchase intention of sustainable EEE.

### 2.1. Attitude and Behavioural Intention towards E-Waste Management

The ability to respond consistently favourably or unfavourably to a particular thing is known as attitude and is the first determinant of TPB framework [38]. A person’s attitude shows whether they perform the appropriate behaviour or not [39]. Evaluating the pros and cons of a behaviour is a part of attitude, which influences behavioural intention [40]. Pertaining to the relationship between intention and behaviour, attitude is crucial. It would not be factually incorrect to say that the intention takes place sometime between weighing your options and actually making the purchase if we understand the intention concept from the perspective of the client and consumer [41]. Thus, the following hypothesis was then put forth:

**H1:**  *The attitudes of a consumer significantly and positively influence behavioural intention towards e-waste management*.

### 2.2. Subjective Norms and Behavioural Intention towards E-Waste Management

The widely perceived societal pressure to engage in or refrain from a behaviour is termed a subjective norm [39]. Subjective Norms are the opinions of whether or not most people agree with the behaviour [42]. Parents’ deeply ingrained family norm values and purchase intentions have been suggested to be linked [28]. People who experienced more social pressure from significant others were observed as more prepared to be green, and as a result, they are more likely to purchase green products [43]. In a study conducted at the European Union level comprising all 28 member countries that examined green product purchasing behaviour, the findings suggested that subjective norms strongly influence green buying behaviour in almost all the countries [44]. Keeping in mind the importance of subjective norms in behavioural intention, the following hypothesis was then put forth:

**H2:**  *The subjective norms of a consumer significantly and positively influence behavioural intention towards e-waste management*.

### 2.3. Perceived Behaviour Control (PBC) and Behavioural Intention towards e-Waste Management

The ability to overcome challenges and carry out the behaviour is referred to as perceived behavioural control [45]. It implies how individuals behave on the basis of prior experiences, resources available and opportunities or obstacles, that he/she is facing [26]. Maichum, Parichatnon and Peng [28] described that purchase intentions in green hotels, organic foods, and green products have been linked to perceived behavioural control. In developed countries, there is a strong link between perceived behavioural control and the intention to separate household waste [46]. PBC shows significant relationship with behavioural intention in previous studies carried out across contexts and countries, such as studies of smartphone waste in Indonesia [34], and consumers’ green purchase behaviour in a developing nation [47,48,49]. Taking cue from other studies, the following hypothesis has been proposed:

**H3:**  *The perceived behaviour control of a consumer significantly and positively influences behavioural intention towards e-waste management*.

### 2.4. Government Policy and Behavioural Intention towards e-Waste Management

To make sustainable consumption apparent to the public in all public buildings and transportation, the government should develop a strategic set of procurement priorities [50]. When purchasing an electronic device, consumers must pay a tax that will be used to offset the expenses of recycling in the future [51,52]. In several industries, laws and regulations require manufacturers to set up product regeneration and appropriate waste management systems. One of the most important factors in motivating sustainable consumption is adherence to regulations [34]. However, expecting state governments to solve long-term sustainability concerns is impractical, particularly in high-risk nations where sustainability policy lacks consistency and/or efficacy [53,54]. Keeping in mind the importance of government policy in previous research, the following hypothesis has been proposed:

**H4:**  *Government policy significantly and positively influences behavioural intention towards e-waste management*.

### 2.5. Financial Benefits and Behavioural Intention towards E-Waste Management

Consumer purchasing power, i.e., financial support/affordability supports environmental awareness, makes a nation a potential market for products that are sustainable [55]. In comparison to the organized industry, informal dealers repair and sell computers, even if they are categorized as e-waste, including some parts that are in functional order [56,57]. Selling any functional portion of a computer would be more profitable than selling it as metal bits [56]. If customers get a discount coupon as a financial benefit to recycle their products as exchange offers offered by sellers of electronics (e.g., Flipkart and Amazon), consumers will become motivated by the financial incentive to engage in sustainable consumption. Hence, the consumers indirectly get themselves involved in e-waste management. Keeping in mind the role of financial benefits, the following hypothesis has been proposed:

**H5:**  *The financial benefits to a consumer significantly and positively influence behavioural intention towards e-waste management*.

### 2.6. Awareness and Behavioural Intention towards E-Waste Management

One relevant issue that has been highlighted is consumers’ unawareness of e-waste recycling and the negative environmental impact of such waste [5]. In a previous study, it was concluded that consumers’ willingness to pay for a product is highly influenced by their awareness of the impact on the environment [58]. Awareness programs that focus on the hazardous consequences of e-waste should be put in place for both manufacturers and consumers, in addition to programs to promote recycling among individuals and businesses. Customers who have proper knowledge about the effects of e-wastes are more likely to not only put in the relevant level of effort but also to spread the word about the hazards of e-waste while encouraging alternative means of disposal [23]. Consumers believe that buying green items is a socially unacceptable behaviour since “significant others” are not fully aware of the benefits of doing so. Thus, governments must educate consumers about the need for e-waste management through various awareness initiatives [35]. Taking cues from previous research, we proposed the following hypothesis:

**H6:**  *The awareness of a consumer significantly and positively influences behavioural intention towards e-waste management*.

### 2.7. Environmental Concern and Behavioural Intention towards E-Waste Management

People who are interested in ecology are inspired by the notion that what they are doing is useful and beneficial to society [59]. Environmental concern is people’s awareness of environmental issues and willingness to fix environmental problems [60]. E-waste is a serious health and environmental issue, especially in Asian countries. Therefore, e-waste management and eco-remediation technologies are necessary. The ever-increasing volume of e-waste poses a great concern to emerging countries such as China and India, where local people and the environment are at risk [61]. Various studies confirm the impact of environmental concern on consumers’ behavioural intentions to choose eco-friendly items and solutions [62,63,64]. Toxic components in e-waste are not only dangerous to the environment, but also to individuals [65]. Thus, the following hypothesis was proposed:

**H7:**  *The environmental concern of a consumer significantly and positively influences behavioural intention towards e-waste management*.

## 3. Research Methodology

The proposed model and the hypotheses were tested by collecting and analysing survey responses using structured questionnaires.

### 3.1. Questionnaire Design

The structured questionnaire used for the survey had two sections. The first section was devoted to the demographic profile of respondents pertaining to gender, age, educational qualification and occupation. The second section consisted of questions related to the TPB constructs (Attitude, Subjective Norms & Perceived Behaviour Control) and extended constructs (Government Policy, Environmental Concern, Financial Benefits and Awareness). A five-point Likert-type scale (1 = Strongly Disagree, 2 = Disagree, 3 = Neutral, 4 = Agree, 5 = Strongly Agree) was used to measure the responses to the constructs. The scale items used in the study have been adapted from the extant literature: Subjective Norms [29,66], Attitude & Behavioural Intention [67], Awareness and Environmental Concern [5]. We developed the items concerning government policy and financial benefits.

### 3.2. Data Collection

We collected data using a Google Form which was sent to the respondents via different social media platforms like WhatsApp (both individuals and groups), Instagram, LinkedIn as well as through E-mails. We were able to generate 524 responses. The demographic profile of the respondents is displayed in Table 1. Out of these 524 responses, 64 responses had to be removed because of incomplete responses and other issues. Thus, a total of 460 responses were found to be fit and usable for the study.

### 3.3. Data Analysis

Partial least square structural equation modelling (PLS-SEM) was used to validate the questionnaire constructs and confirm the relationships among the variables [29]. SEM was conducted by SMART PLS 3.0 [68] which evaluated the hypothesized conceptual model of this study. SEM consists of two components: One is a measurement model and other is a structural model. The measurement model (AKA Outer model) assesses the reliability and validity of latent (unobserved variables) as a linear function of indicators (observed variables). The structural model (AKA Inner Model) shows the direction and strength of the relationship between variables [69].

#### Measurement Model Analysis

**Figure 2:**
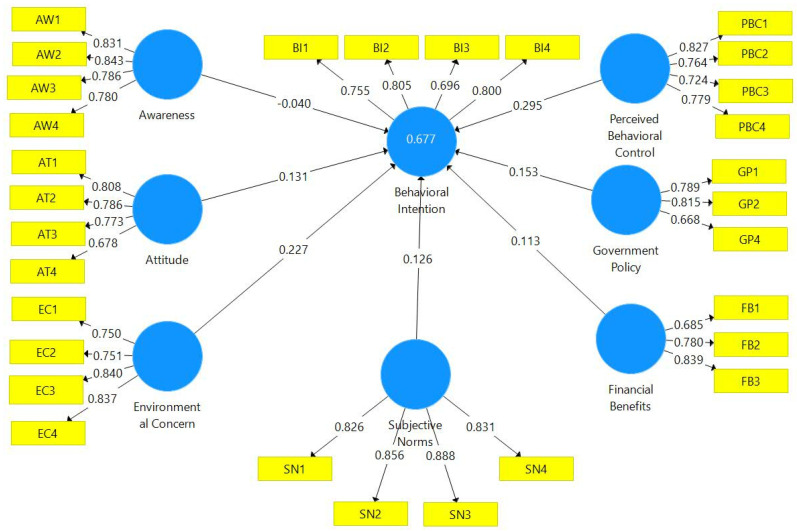
Loadings on measurement model.

To check the reliability level of the instrument, we use the Cronbach’s Alpha. If the value falls within the range of 0.61–0.70, it would be considered as moderate and acceptable; if the value falls between 0.71–0.80, it is considered as good and acceptable; if the value is between 0.81–0.90, it is considered good, and a value between 0.91–1.00 is considered excellent [70,71,72]. In the case of Composite or construct reliability the value should be greater than 0.70 [73]. The findings suggest the constructs in this study fulfil this criterion (Table 2*)*. Convergent validity was confirmed after examining the factor loadings (Figure 2) and average variance extracted (AVE) > 0.50. In Table 2, the values of Cronbach’s alpha and Composite reliability are within the acceptable limits. Therefore, there is sufficient consistency of constructs.

Table 3, Table 4 and Table 5 show the discriminant validity using Fornell-Larcker criterion, cross-loadings and Heterotrait–Monotrait (HTMT) ratio. According to the Fornell-Larcker criterion, the square root of AVE should be greater than the correlation values of other constructs [74]. In Table 3, the values on the diagonal (bold) reflect the square root of the AVE, whereas the values of the diagonal are correlations. Therefore, it can be said that discriminant validity is established as per this criterion.

According to cross-loadings, a specific item should have higher loadings on its own parent construct than other constructs in the study. In Table 4, cross-loadings justified the discriminant validity. The third and last step to measure discriminant validity is the HTMT (Heterotrait–Monotrait) ratio, which suggests a threshold value of 0.90 [68]. All HTMT ratios are below 0.90 (Table 5); hence, discriminant validity is established to determine the distinctiveness of the constructs in the study.

### 3.4. Structural Model

As per the suggestions of Hair, Hult, Ringle and Sarstedt [73], the hypothesized relationships and correlations between variables were verified using a bootstrapping procedure with sub-samples of 5000. The structural model assessment technique evaluates: (1) construct collinearity; (2) path coefficient significance; (3) coefficient of determination (R^2^) level; (4) effect magnitude (f^2^); and (5) predictive relevance (Q^2^).

*Construct Collinearity*—The variance inflation factor (VIF) values were found to be less than 3 (Table 6) which shows no concern of multi-collinearity between latent variables.

*Path Coefficient Significance*—The hypotheses were tested using path coefficients and t-values. Out of the seven hypotheses, six hypotheses (H_1_–H_5_, and H_7_) were accepted as the t-values were greater than 1.96 and the significance level was less than 0.05 [75] and H_6_ (relationship between awareness and behavioural intention) could not be accepted. The findings of the path coefficients are presented in Table 7.

**Figure 3:**
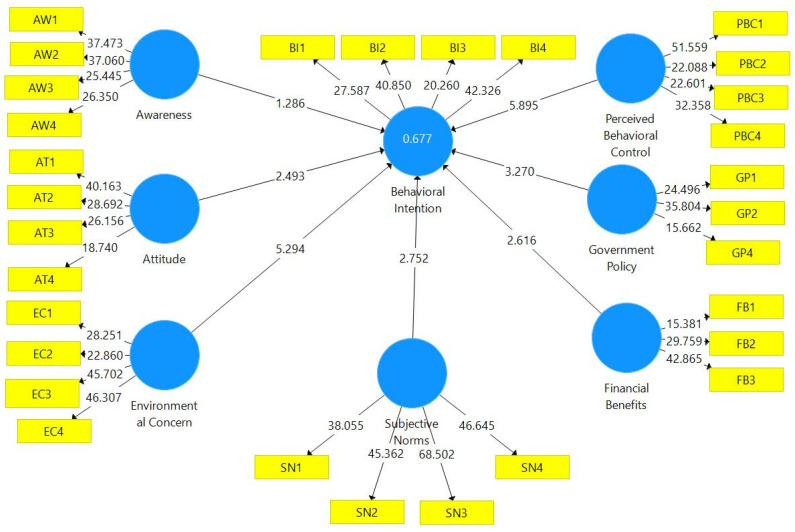
Structural Model.

*Coefficient of Determination*—As suggested by Hair et al. [76], coefficient of determination values (R^2^): 0.75, 0.50 or 0.25 can be, respectively described as substantial, moderate or weak. The R^2^ value for the present model has been found to be 0.677 (Figure 3) which describes variance to be substantial.

*Effect Magnitude*—Researchers have suggested the (Effect Magnitude) f^2^ values near 0.02, 0.15, and 0.35 represents weak, moderate, and large effects, respectively [73,77]. The effect magnitude of the model indicates how much an independent latent variable contributes to the R^2^ of a dependent latent variable. In other words, effect size evaluates the strength of the relationship between the latent variables. The results (Table 8) revealed that the f-square effect size for the present model ranged from 0.003 (negligible) to 0.109 (moderate).

*Predictive Relevance*—Predictive relevance, i.e., Q^2^ evaluates cross-validated redundancy to assess the inner model. It measures whether a model has predictive relevance or not (greater than zero is good). The Q^2^ value of the present model was found to be 0.387 which established the predictive relevance of the model.

## 4. Discussion and Conclusions

As environmental degradation is a serious concern that the world is facing, the present study aims to contribute to the research literature on this topic. Several researchers have used the TPB model to study sustainable behaviour in different contexts. In the current study, we have tried to explore the sustainable behaviour of consumers concerning e-waste management. To cope up with previous criticisms of TPB [5,55,63,65] of not comprising several important factors, in the present work we have extended the TPB model including four new variables. The findings of the present study could be useful to different stakeholders in society. The results suggest that behavioural intention for young consumers to manage e-waste can be predicted by attitude, subjective norms, perceived behavioral control, government policy, financial benefits, awareness as well as environmental concern. All of the variables except awareness have been proven to impose significant effects on behavioural intention of e-waste management. For example, government policies have been found to have a positive and significant influence on behavioural intentions. Thus, it can be surmised that government should come up with policies that motivate people to think of e-waste management. They need to focus on spreading awareness through educational institutions and programs. An illustrative example is an initiative by the Delhi government to reduce pollution by providing an e-waste facility through an eco-park for the safe handling and disposal of e-waste [78].

The study findings suggest that young consumers are highly aware of e-waste management. Despite this, the generation of e-waste generation is still at a peak level. Different studies have shown a positive impact of awareness on e-waste management [5,79,80] but the findings of the present study suggest no such impact of awareness on e-waste management, similar to the findings of Kumar [29]. Paradoxically, based on the findings, it can be argued that consumers are aware but do not consider behaving in an environmentally friendly way. The study findings also showed financial benefits to positively impact the behavioural intentional towards e-waste management. Thus, marketers and the government need to build effective strategies that can motivate consumers to participate in e-waste management. Marketers should come up with attractive offers like value exchange offers, selling refurbished devices at a good price, and selling recycled products at a low price. The government, on the other hand, develops policies to help marketers to execute their plans. The study reflects environmental concern among people positively and significantly influences behavioural intention towards e-waste management. 

The extant literature suggests a high generation of e-waste, out of which only a bit of waste is being managed. It is therefore of utmost importance that all the stakeholders cooperate to make the consumers aware of the perils of e-wastes and mould them towards environmental concerns. In the long run, this can help countries around the world manage e-wastes in a better way. The findings of the present study suggest that government policy, financial benefits, and environmental concerns play a relevant role in shaping the sustainable behavioral intention of young consumers. It can be surmised that the inclusion of the above three factors in the TPB makes it a robust model with respect to e-waste management. The study findings may be helpful to the academic community by suggesting a model that may be tested in different settings. Marketers can formulate strategies keeping in mind the study findings, whereby they can consider both profitability and the environment. The government may also use the findings of the study to formulate policies keeping in mind the sustainability of the environment. Finally, the study findings could help readers to understand the importance of e-waste management in our day-to-day life.

## 5. Limitations and Scope for Future Research

The study has certain limitations that can be addressed in future studies. In the present study, we have focused on young consumers mainly from Delhi NCR. In the future, researchers may study the same constructs with consumers of different regions of this country. While this research has taken waste management of electronic and electrical equipment into consideration, future researchers may consider other product categories as well. Another issue is related to the educational level of the respondents. In this study, only educated respondents were considered, but in the future studies can be done on uneducated people as well. It is also possible that more novel insights could be found by involving samples from a wider demographic population comprising different cultures. Finally, researchers could employ a longitudinal approach, which would allow for examining evolution and changes in the above-mentioned factors and behavioral intentions.

## Figures and Tables

**Table 1:** Demographics of 460 respondents.

	Frequency
*Gender*	
Male	218
Female	242
*Educational Qualification*	
Up to Senior Secondary	86
Under-Graduate	190
Post-Graduate	157
PhD	27
*Occupation*	
Student	258
Businessman	47
Employed	123
Homemaker	22
Others	10

**Table 2:** Construct Reliability and Convergent Validity.

	Cronbach’s Alpha	Composite Reliability	AVE
Attitude	0.761	0.847	0.582
Awareness	0.833	0.884	0.657
Behavioural Intention	0.764	0.849	0.585
Environmental Concern	0.807	0.873	0.633
Financial Benefits	0.657	0.814	0.594
Government Policy	0.636	0.803	0.577
Perceived Behavioural Control	0.779	0.857	0.6
Subjective Norms	0.872	0.913	0.724

**Table 3:** Fornell-Larcker Criterion.

	AT	AW	BI	EC	FB	GP	PBC	SN
Attitude (AT)	**0.763**							
Awareness (AW)	0.481	**0.811**						
Behavioural Intention (BI)	0.675	0.409	**0.765**					
Environmental Concern (EC)	0.708	0.341	0.694	**0.796**				
Financial Benefits (FB)	0.472	0.347	0.584	0.58	**0.771**			
Government Policy (GP)	0.589	0.355	0.637	0.609	0.577	**0.76**		
Perceived Behavioural Control (PBC)	0.655	0.533	0.71	0.58	0.501	0.541	**0.774**	
Subjective Norms (SN)	0.526	0.469	0.591	0.466	0.447	0.469	0.637	**0.851**

Note: The values in bold reflect the square root of the AVE which should be greater than the correlation values of other constructs.

**Table 4:** Cross Loadings.

	AT	AW	BI	EC	FB	GP	PBC	SN
AT1	**0.808**	0.319	0.598	0.639	0.425	0.523	0.52	0.446
AT2	**0.786**	0.48	0.493	0.51	0.396	0.397	0.505	0.426
AT3	**0.773**	0.232	0.524	0.631	0.374	0.487	0.478	0.338
AT4	**0.678**	0.477	0.425	0.336	0.218	0.37	0.505	0.399
AW1	0.503	**0.831**	0.428	0.438	0.374	0.391	0.464	0.44
AW2	0.417	**0.843**	0.346	0.289	0.311	0.285	0.475	0.311
AW3	0.279	**0.786**	0.24	0.101	0.175	0.192	0.381	0.335
AW4	0.274	**0.78**	0.245	0.159	0.192	0.213	0.381	0.43
BI1	0.51	0.271	**0.757**	0.485	0.401	0.447	0.521	0.382
BI2	0.529	0.316	**0.806**	0.578	0.467	0.507	0.58	0.5
BI3	0.419	0.295	**0.694**	0.411	0.423	0.399	0.483	0.404
BI4	0.59	0.364	**0.798**	0.623	0.492	0.576	0.58	0.509
EC1	0.585	0.282	0.532	**0.75**	0.442	0.513	0.47	0.365
EC2	0.434	0.249	0.452	**0.751**	0.423	0.429	0.383	0.256
EC3	0.575	0.256	0.575	**0.84**	0.484	0.487	0.471	0.38
EC4	0.634	0.297	0.628	**0.837**	0.492	0.503	0.507	0.454
FB1	0.238	0.229	0.38	0.38	**0.685**	0.417	0.311	0.274
FB2	0.333	0.337	0.454	0.408	**0.78**	0.371	0.43	0.397
FB3	0.491	0.238	0.508	0.541	**0.84**	0.539	0.41	0.356
GP1	0.475	0.32	0.53	0.469	0.527	**0.788**	0.461	0.39
GP2	0.445	0.231	0.527	0.503	0.415	**0.816**	0.43	0.338
GP4	0.428	0.262	0.377	0.411	0.363	**0.668**	0.327	0.347
PBC1	0.648	0.406	0.667	0.577	0.441	0.455	**0.827**	0.536
PBC2	0.44	0.287	0.498	0.466	0.45	0.502	**0.764**	0.456
PBC3	0.36	0.453	0.43	0.257	0.293	0.304	**0.724**	0.439
PBC4	0.525	0.513	0.563	0.44	0.353	0.401	**0.779**	0.53
SN1	0.452	0.413	0.493	0.371	0.336	0.352	0.522	**0.826**
SN2	0.465	0.374	0.472	0.377	0.356	0.403	0.482	**0.856**
SN3	0.454	0.378	0.504	0.452	0.431	0.417	0.567	**0.888**
SN4	0.422	0.427	0.537	0.382	0.393	0.422	0.589	**0.831**

Note: The values in bold show the loadings of the parent construct on its own construct; it should always be greater than loading on other constructs.

**Table 5:** Heterotrait–Monotrait Ratio (HTMT).

	AT	AW	BI	EC	FB	GP	PBC	SN
Attitude								
Awareness	0.586							
Behavioural Intention	0.87	0.48						
Environmental Concern	0.876	0.368	0.865					
Financial Benefits	0.639	0.437	0.818	0.788				
Government Policy	0.841	0.457	0.892	0.846	0.882			
Perceived Behavioural Control	0.83	0.65	0.899	0.703	0.69	0.752		
Subjective Norms	0.648	0.543	0.717	0.545	0.586	0.632	0.764	

**Table 6:** VIF (Variance Inflation Factor).

AT1	1.565	EC4	1.763
AT2	1.593	FB1	1.201
AT3	1.484	FB2	1.327
AT4	1.336	FB3	1.422
AW1	1.619	GP1	1.255
AW2	1.895	GP2	1.338
AW3	2.368	GP4	1.199
AW4	2.293	PBC1	1.614
BI1	1.51	PBC2	1.54
BI2	1.627	PBC3	1.478
BI3	1.34	PBC4	1.531
BI4	1.515	SN1	1.986
EC1	1.476	SN2	2.284
EC2	1.626	SN3	2.66
EC3	1.914	SN4	1.918

**Table 7:** Path Coefficients.

		T Statistics	*p* Values	Hypothesis
H1	Attitude → Behavioural Intention	2.47	0.014	Accepted
H2	Subjective Norms → Behavioural Intention	2.723	0.006	Accepted
H3	Perceived Behavioural Control → Behavioural Intention	5.847	0	Accepted
H4	Government Policy → Behavioural Intention	3.26	0.001	Accepted
H5	Financial Benefits → Behavioural Intention	2.58	0.01	Accepted
H6	Awareness → Behavioural Intention	1.255	0.21	Failed to Accept
H7	Environmental Concern → Behavioural Intention	5.19	0	Accepted

**Table 8:** F-square.

	F-SQUARE	EFFECTS
Attitude	0.02	Weak
Awareness	0.003	Negligible
Environmental Concern	0.063	Weak
Financial Benefits	0.022	Weak
Government Policy	0.036	Weak
Perceived Behavioural Control	0.109	Moderate
Subjective Norms	0.026	Weak
